# Development of a Dual ELISA for the Detection of CD2v-Unexpressed Lower-Virulence Mutational ASFV

**DOI:** 10.3390/life11111214

**Published:** 2021-11-10

**Authors:** Changjie Lv, Ya Zhao, Lili Jiang, Li Zhao, Chao Wu, Xianfeng Hui, Xiaotong Hu, Ziqi Shao, Xiaohan Xia, Xiaomei Sun, Qiang Zhang, Meilin Jin

**Affiliations:** 1 State Key Laboratory of Agricultural Microbiology, Huazhong Agricultural University, Wuhan 430070, China; lvcj@webmail.hzau.edu.cn (C.L.); zhaoyafeiba@webmail.hzau.edu.cn (Y.Z.); jll123456@webmail.hzau.edu.cn (L.J.); 202021107011152@stu.hubu.edu.cn (L.Z.); zhangjiali0819@webmail.hzau.edu.cn (C.W.); huixianfeng@webmail.hzau.edu.cn (X.H.); hu_xiaotong@webmail.hzau.edu.cn (X.H.); 202021107011114@stu.hubu.edu.cn (X.X.); xiaomei@webmail.hzau.edu.cn (X.S.); 2 College of Veterinary Medicine, Huazhong Agricultural University, Wuhan 430070, China; 2019302220411@webmail.hzau.edu.cn; 3 College of Biomedicine and Health, Huazhong Agricultural University, Wuhan 430070, China

**Keywords:** African swine fever virus (ASFV), ELISA, differentiation, non-hemadsorption, lower-virulence natural mutants

## Abstract

African swine fever virus (ASFV) is an important viral pathogen infecting pigs worldwide throughout the pig industry. CD2v (an outer-membrane glycosylated protein of ASFV)-unexpressed lower-virulence mutants have appeared in China and other countries in recent years. Using OIE-recommended quantitative PCR and ELISA methods, people can accurately judge whether pigs are infected with wild-type ASFV. However, the strategy has failed to distinguish ΔCD2v lower-virulence mutants and wild-type ASFV infection. Here, we expressed and purified the CD2v and p30 proteins via CHO cells and successfully established a dual enzyme-linked immunosorbent assay (ELISA), which can be used to differentiate pigs infected with wild-type ASFV or with CD2v-unexpressed lower-virulence mutants. The dual ELISA showed excellent specificity without cross-reactions with antibodies of PRRSV, CSFV, JEV, PRV, or PPV. The dual ELISA could detect ASFV-infected positive serum samples up to dilutions of 5120 times, possessing high sensitivity. Therefore, the application of this dual ELISA approach can play an important role in ASFV epidemiology study and fill the gaps in differential diagnosis.

## 1. Introduction

African swine fever (ASF), a highly contagious viral disease. It infects wild boars and domestic pigs of all ages, and its high mortality rate and rapid spread causes enormous economic damage for pig production worldwide [1,2]. ASF is caused by African swine fever virus (ASFV), which belongs to the genus *Asfivirus* of the *Asfarviridae* family [3]. The full length of the ASFV DNA genome varies between 170 kb and 190 kb, and it contains 151 to 167 genes depending on the different strains [4]. On the basis of the ASFV B646L gene, which encodes the p72 major capsid protein, ASFV can be divided into 23 diverse genotypes [5]. At present, there are no effective and approved vaccines and medicines for ASFV.

In 1921, ASFV was originally reported in Kenya, Africa. It was detected outside the African continent for the first time in Portugal in 1957, followed by Spain, Italy, France, and the Iberian Peninsula in the mid-1990s [6]. In 2007, there was further transcontinental spread of ASF as it was introduced into Georgia in the Caucasus region and, from there, spread to neighboring countries including Armenia, Azerbaijan, and the Russian Federation [7]. In 2018, an initial outbreak of ASF in pigs was reported in China, which is the world’s biggest pig producer and pork consumer [8]. On the basis of its p72 protein sequence, this ASFV strain is classified as genotype II. The disease has spread to 23 provinces in China, causing 182 outbreaks in China (till 25 October 2021). ASF has led to the death of more than 1.2 million pigs and an estimated loss of more than 240 million USD to the Chinese economy. Although tremendous efforts were taken to combat the disease, including laboratory testing, slaughter of infected and susceptible pigs, and application of strict sanitary and biosecurity measures, ASF still poses economic losses in the country.

In recent years, novel CD2v-unexpressed lower-virulence natural ASFV mutants have appeared [9,10] and even become epidemic in China [9,11]. In China, 22 ASFV strains are isolated from a total of 3660 samples, and 11 isolates are CD2v-unexpressed lower-virulence natural mutants accounting for 50% [9,11]. The amino acid substitutions, deletions, and insertions of wild-type ASFV CD2v are the main characteristics of ASFV-ΔCD2v, leading to the early termination of translation and frameshift mutations, thereby resulting in a non-hemadsorption (non-HAD) phenotype in infected macrophages [11]. Previous research found that pigs infected with CD2v-unexpressed lower-virulence natural mutants can shed the virus, and display disease signs including arthroncus, phyma, cutaneous necrosis, and depression, lacking typical clinical symptoms of wild-type ASFV infection [9,11]. When using the OIE-recommended quantitative PCR (qPCR) and ELISA, it is harder to detect CD2v-unexpressed lower-virulence natural mutants compared to the typical highly lethal strains in oral, rectal, and blood samples, but not in the mediastinal lymph node, causing greater challenges for ASF control [4,11]. Thus, there is an urgent need to develop a convenient, high-sensitivity detection method that distinguishes CD2v-unexpressed lower-virulence natural mutants and wild-type ASFV infections.

In this study, we established a dual ELISA approach that distinguishes CD2v-unexpressed lower-virulence natural mutants and wild-type ASFV. The use of this dual ELISA can determine whether pigs infected with CD2v-unexpressed lower-virulence natural mutants or wild-type ASFV, and plays an important role in epidemiological study of ASFV.

## 2. Materials and Methods

### 2.1. Swine Serum and Secondary Antibody

Swine serum samples (*n* = 10) of a CD2v-unexpressed strain of ASFV (ASFV-ΔCD2v) and swine serum samples (*n* = 10) of a virulent ASFV strain (wild-type ASFV) were stored at −80 °C until use. Swine serum samples (*n* = 43) were also collected from pig farms. Furthermore, serum samples (*n* = 20) positive for classical swine fever virus (CSFV), porcine reproductive and respiratory syndrome virus (PRRSV), Japanese encephalitis (JEV), pseudorabies virus (PRV), and porcine parvovirus (PPV) infection were conserved in our laboratory. A commercial horseradish peroxidase (HRP)-conjugated affinipure goat anti-swine IgG (H + L) secondary antibody was used at a dilution of 1:30,000 (Proteintech, Wuhan, China).

### 2.2. Eukaryotic Expression and Purification of CD2v and p30 Proteins

The N-terminal 1–205 amino acids of ASFV CD2v and the full-length p30 (GenBank accession No. MK333180.1) were synthesized and inserted into the pXC17.4 vector, thereby resulting in the construction of the recombinant plasmids pXC17.4-CD2v and pXC17.4-p30. The plasmids were transfected into Chinese hamster ovary (CHO) cells using Lipofectamine 3000 transfection reagent according to the procedure recommended by the manufacturer. Protein purification was performed using Ni-NTA agarose (GE Healthcare, Pittsburgh, PA, USA) on an AKTA FPLC (GE Healthcare, Pittsburgh, PA, USA) according to the instruction manual.

### 2.3. Western Blotting Analysis

For western blotting analysis, p30 and CD2v proteins were analyzed by 12% sodium dodecyl sulfate-polyacrylamide gel electrophoresis (SDS-PAGE) and transferred onto pure nitrocellulose membranes (GE, Boston, MA, USA), which was blocked with 1% BSA in TBST at room temperature for 2 h, and then incubated with ASFV-positive or -negative serum as primary antibody at 4 °C overnight. After incubating with HRP-conjugated anti-pig secondary antibodies for 1 h at room temperature, signals were visualised by ECL reagent (Advansta, San Jose, CA, USA).

### 2.4. Establishment of Enzyme-Linked Immunosorbent Assay (ELISA) and ELISA Kit

A commercially available ASFV VP72 antibody blocking ELISA kit was purchased from Ingenasa (INgezim 11.PPA.K3, Madrid, Spain). First, 96-well microtitration plates (Thermo Fisher Scientific, Waltham City, MA, USA) were manually coated with ASFV CD2v or p30 proteins at a concentration of 100 ng or 80 ng per well. ELISA conditions, e.g., CD2v and p30 coating conditions, sample and secondary antibody dilutions, and incubation times, were concurrently optimized for the antibody detection in swine serum. The experimental procedure was performed as previously reported [12]. Briefly, serum samples were diluted 1:40, after which plates were loaded with 100 μL of diluted sample per well. Antibody-positive and -negative controls were added to each ELISA plate. Plates were incubated at 37 °C for 30 min and then washed five times. Thereafter, 100 μL of horseradish peroxidase-conjugated goat anti-swine IgG (H + L) antibody diluted 1:30,000 was added to each well, and the plates were incubated at 37 °C for 30 min. After a washing step, the peroxidase reaction was visualized by adding 100 μL of tetramethylbenzidinehydrogen peroxide (TMB) substrate solution per well. Plates were incubated at room temperature for 15 min, 50 μL of stop solution was added, and the plates were read immediately thereafter. Reactions were measured as a function of optical density (OD) at 630 nm using an ELISA plate reader (Bio-Tek, Winusky, VT, USA). According to a previously established protocol [13,14,15], sera were considered positive if the OD values were twofold higher than the mean OD of the 43 sera collected from farms and conserved in our laboratory.

### 2.5. Statistical Analysis

All the experiments were repeated at least three times. The GraphPad Prism 5.0 software was used to perform all the statistical testing, as detailed in the figure legends. All data were presented as the mean ± SD. Significant differences between samples were assessed using Student’s *t*-test.

## 3. Results

### 3.1. Expression of ASFV CD2v and p30 and Immunogenic Identification

**Figure 1:**
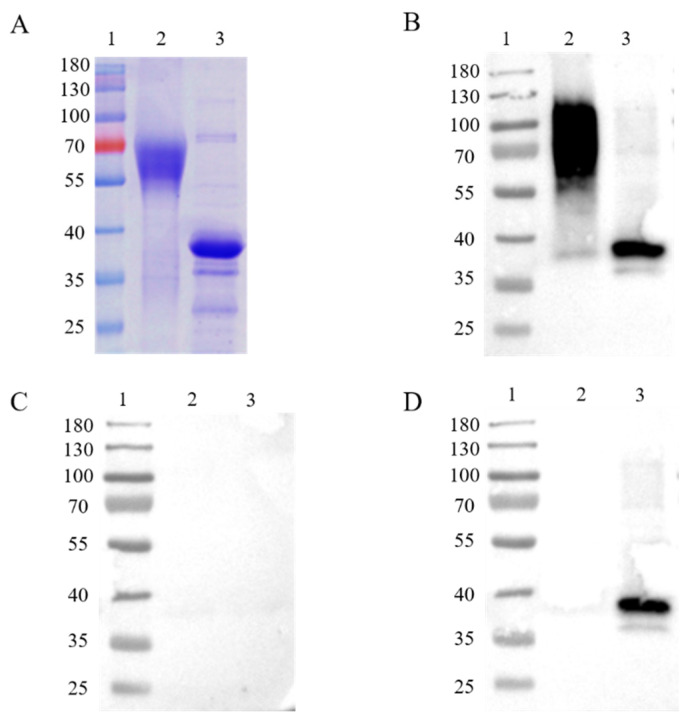
SDS-PAGE and western blot analysis of purified recombinant CD2v (63 kDa) and p30 (35 kDa) proteins (**A**). Analysis of immunogenic identification of p30 and CD2v proteins by western blot assay (**B**–**D**). Recombinant CD2v and p30 proteins could react with ASFV-positive sera (**B**), but not-negative sera (**C**). The recombinant p30 protein could react with ASFV-ΔCD2v-infected sera, in contrast to the CD2v protein (**D**). Lane 1: protein marker; Lane 2: CD2v; Lane 3: p30.

In order to express intact, full-length proteins, Chinese hamster ovary (CHO) cells, a eukaryotic expression cell line, were used to produce the ASFV CD2v and p30 proteins. The recombination protein was successfully acquired in CHO cells. A molecular weight of about 63 kDa on the SDS-PAGE gel was shown on account of glycosylation modifications of the CD2v protein in mammalian cells (Figure 1A), while the p30 protein was displayed at a molecular weight of about 35 kDa (Figure 1A). Western blot analysis was applied for the immunogenic identification of CD2v and p30 via CHO cell expression. The recombinant CD2v and p30 proteins could react with ASFV-positive but not -negative serum (Figure 1B,C). The recombinant p30 proteins, but not CD2v proteins, could react with ASFV-ΔCD2v-positive serum (Figure 1D).

### 3.2. Optimization of Experimental Conditions for ELISA

In the ELISA assay, six experimental conditions were optimized, namely the concentration of CD2v and p30, reaction time, dilution of HRP-labeled secondary antibody and serum samples, and reaction time of TMB substrate solution. The ratio of ASFV-positive and -negative serum (P/N) was analyzed by phalanx titration for the optimization of reaction conditions.

**Figure 2:**
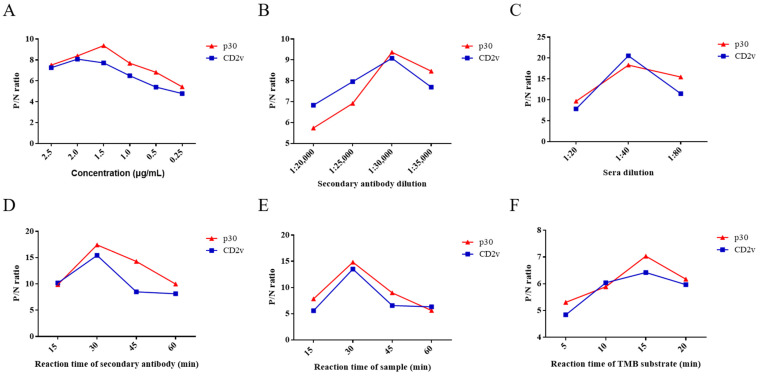
Optimization of ELISA conditions for antibody detection of ASFV CD2v or p30. P/N ratios of an ASFV-positive and -negative serum using different concentrations of CD2v and p30 (**A**). P/N ratios of an ASFV-positive and -negative serum using HRP-labeled secondary antibody of different concentrations for CD2v and p30 (**B**). P/N ratios of an ASFV-positive and -negative serum with various dilutions of serum samples and various reaction times of the HRP-labeled secondary antibody (**C**,**D**). P/N ratios of an ASFV-positive and -negative serum with various reaction times of serum samples and TMB substrate solution, respectively (**E**,**F**). Data are shown as the mean ± SD. Blue lines with squares indicate CD2v, whereas red lines with triangles indicate p30.

As the results show, when the concentration of CD2v and p30 proteins was 2 μg/mL and 1.5 µg/mL, respectively, they displayed the highest P/N ratio by phalanx titration (Figure 2A). Therefore, the optimum concentration of ELISA kit using CD2v or p30 proteins as coating antigens was 2 μg/mL or 1.5 µg/mL. When dilution of HRP-labeled secondary antibody and serum samples was 1:30,000 and 1:40, respectively, which achieve the highest P/N ratio (Figure 2B,C). The optimized reaction times of the HRP-labeled secondary antibody, serum samples, and TMB substrate solution were 30, 30, and 15 min, respectively (Figure 2D–F). Ultimately, these six optimized conditions were used in subsequent assays.

### 3.3. Determination of the Cut-off Value of ELISA

**Figure 3:**
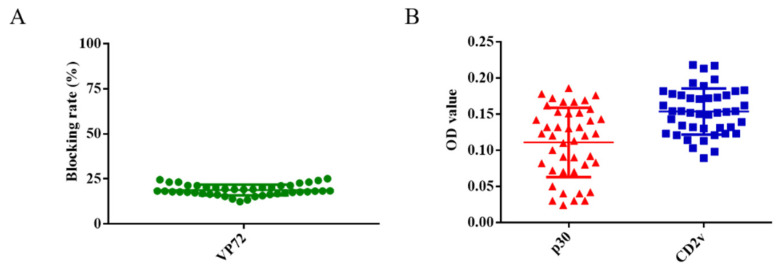
Determination of the cut-off values for CD2v and p30 ELISA. A total of 43 serum samples were detected using the OIE-recommended INgezim ASFV antibody blocking ELISA kit (**A**). These serum samples were also detected using the newly established ELISA for CD2v and p30 proteins (**B**).

A total of 43 swine serum samples were used to determine the cut-off value of the ELISA. Firstly, these serum samples were detected using the OIE-recommended INgezim ASFV antibody blocking ELISA kit according to the manufacturer’s instructions, which yielded negative results (Figure 3A). These 43 samples were also measured using the newly established dual ELISA for CD2v and p30, showing results consistent with those of the INgezim ELISA kit. The average OD value of these serum samples was 0.16 using the ELISA for CD2v and 0.11 using the ELISA for p30 (Figure 3B). Two times of the average OD value was set as cut-off value according to previously established researches [13,14,15]. Therefore, OD values of 0.32 and 0.22 were established as the cut-off for the CD2v and p30 ELISA, respectively.

### 3.4. Detection Sensitivity and Specificity of ELISA

**Figure 4:**
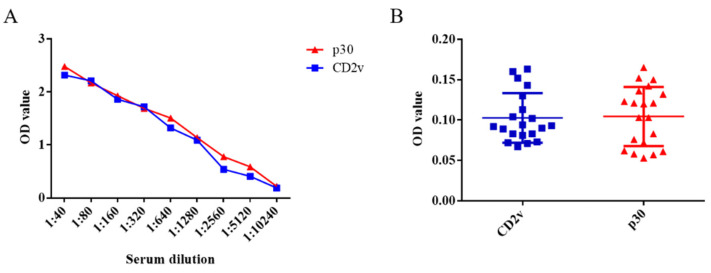
Detection sensitivity and specificity of the ELISA kits based on the CD2v or p30 proteins for ASFV. Ten ASFV-infected serum samples were subject to a series of dilutions. According to the cut-off value, the positivity of these serum samples could be detected up to a dilution of 5120 times (**A**). The two ELISA kits based on CD2v or p30 proteins did not react with 1:40 diluent CSFV-, PRRSV-, JEV-, PRV-, and PPV-infected serum samples, highlighting their specificity (**B**). Data are shown as the mean ± SD. Blue lines with squares indicate CD2v, whereas red lines with triangles indicate p30.

Ten positive serum samples were used in the experiment to test the sensitivity of the dual ELISA, whereby the positivity of these serum samples could be detected up to a dilution of 5120 times (Figure 4A). Moreover, CSFV-, PRRSV-, JEV-, PRV-, and PPV-infected serum samples could not be detected in either case, which illustrates their excellent specificity (Figure 4B). Therefore, both ELISA approaches fully met the characteristics of sensitivity and specificity for the detection of swine serum samples in farms.

### 3.5. Dual ELISA Kit Detection of ASFV- or ASFV-ΔCD2v-Infected Sera

**Figure 5:**
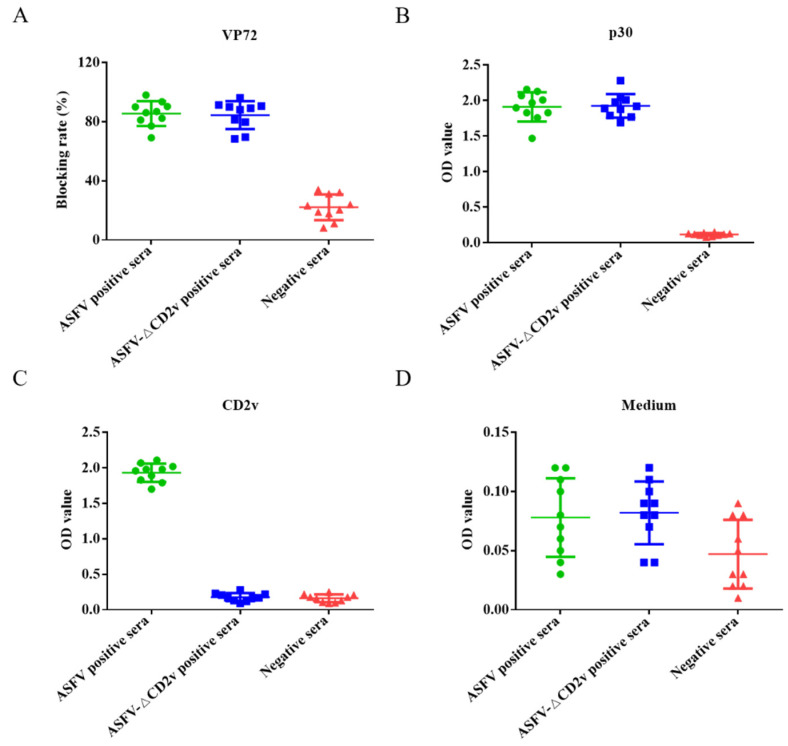
The dual ELISA was used for the detection of ASFV- or ASFV-ΔCD2v-infected serum samples. The INgezim ASFV antibody blocking ELISA kit were used to detect ASFV- and ASFV-ΔCD2v-infected and negative serum samples (**A**). The newly established ELISA based on p30 were used to detect ASFV- and ASFV-ΔCD2v-infected and negative serum samples (**B**). The newly established ELISA based on CD2v were used to detect ASFV- and ASFV-ΔCD2v-infected and negative serum samples (**C**). The ELISA based on medium from CHO cells transfected with empty plasmid were used to detect ASFV- and ASFV-ΔCD2v-infected and negative serum samples (**D**).

The dual ELISA was used to distinguish between ASFV- and ASFV-ΔCD2v-infected swine serum samples. Swine serum samples (*n* = 10) infected with ASFV-ΔCD2v, samples (*n* = 10) infected with wild-type ASFV, and negative serum samples (*n* = 10) were utilized in the assay. When the blocking rate of serum sample is more than 50% using the INgezim ASFV antibody blocking ELISA kit for ASFV antibody detection, the sample is positive according to the manufacturer’s instructions. When the blocking rate of serum sample is less than 40%, the sample is negative. The blocking rates of a total of 20 serum samples from pigs infected by wild-type ASFV or ASFV-ΔCD2v were more than 50%, and blocking rates of 10 negative serum samples were less than 40% using blocking ELISA kit (Figure 5A). The dual ELSIA kits using p30 or CD2v proteins as coating antigens were indirect ELSIA. If OD value of serum samples was more than 0.32 and 0.22, sample was positive using CD2v and p30 ELISA, respectively. The OD values of 20 positive serum samples were more than 0.22, and the OD values of 10 negative serum samples were less than 0.22 using ELISA of p30 as coating antigen (Figure 5B). The OD values of the 10 serum samples from pigs infected with wild-type ASFV were more than 0.32, and OD values of 10 negative serum samples and 10 serum samples from pigs infected with ASFV-ΔCD2v were less than 0.32 using ELISA of CD2v as coating antigens (Figure 5C). We used this ELISA based on medium from CHO cells only transfected with empty plasmid as coating antigen to detect negative control serum, ASFV-positive and -negative serum samples, and all of the test results were negative (Figure 5D). These results showed that both of the two ELISA kits can distinguish between wild-type ASFV-positive and -negative serum samples consistent results with OIE-recommended INgezim ASFV antibody blocking ELISA kit. Furthermore, the dual ELISA kit based on p30 and CD2v proteins can be used to differentiate wild-type ASFV from ASFV-ΔCD2v infection, but the OIE-recommended ELISA kit cannot.

## 4. Discussion

In this study, the ASFV CD2v and p30 proteins were successfully expressed in CHO cells. The two recombinant proteins were used to establish a high-sensitivity and high-specificity dual ELISA, which was utilized to distinguish pigs infected with ASFV-ΔCD2v from those infected with wild-type ASFV. The dual ELISA can play an indispensable role in the detection of different strains and epidemiological study of ASFV.

The full-length glycosylated form of ASFV CD2v is detected in infected cells with a molecular weight of 89 kDa, in addition to an N-terminal glycosylated fragment of 63 kDa and a C-terminal non-glycosylated fragment of 26 kDa [16]. In this study, the N-terminal glycosylated domain (1–205 AA) was expressed using a eukaryotic expression system (Figure 1A). The ELISA kit targeting the glycosylated CD2v protein could distinguish between ASFV-positive and -negative serum samples (Figure 5C). A previous study showed that a glycosylated protein was the most important immunogenic antigen for avian reticuloendotheliosis virus [17]. Therefore, the glycosylated CD2v protein of ASFV may have an important function in infected cells. CD2v, a highly glycosylated outer-membrane protein of ASFV, likely associates with the glycosylated receptors of host cells during ASFV invasion. The expressed CD2v protein can be used to study the interaction between receptors and ligand, the process of which requires further investigation. ASFV p30 is a phosphorylated protein expressed at the early stage in infected cells [18]. ELISA kits based on p30 are widely used to detect ASFV infection [18,19,20]. In this study, the ELISA kit based on p30 via CHO cell expression was able to differentiate ASFV-positive and -negative serum (Figure 5B).

ASFV exhibit hemadsorption (HAD) activity of red blood cells in infected macrophages through the CD2v protein. However, non-expression of this protein can lead to a non-hemadsorption (non-HAD) phenotype [21,22]. Previous studies described the presence of non-HAD genotype I isolates in Portugal as well as non-HAD genotype II isolates in Europe in 2017 [9,10], which could lead to an attenuation of viral virulence. ASFV-ΔCD2v strain has also been detected in China, leading to non-HAD phenotype [11,23]. These CD2v-unexpressed lower-virulence natural mutants cause nonlethal, subacute, or chronic disease, as well as persistent infection; importantly, their qPCR detection based on viral DNA is difficult using oral and rectal swabs from infected pigs owing to irregular virus shedding [11]. The detection of infection with mutants in pigs has caused huge economic losses in farms. Although the OIE-recommended ELISA can be used to detect ASFV antibody, it cannot distinguish wild-type ASFV and CD2v-unexpressed lower-virulence natural mutants. Our dual ELISA was successfully used in this study to distinguish between ASFV and ASFV-ΔCD2v infection (Figure 5B,C). Certainly, ELISA kits targeting other ASFV proteins such as p72 or p54 can also be applied together with the CD2v kit to detect serum samples from farms.

The ELISA method has been widely used for the detection of samples in clinical diagnostic laboratories [24]. Fenton et al. used ELISA to detect border disease virus (BDV) antibody in sheep serum [25]. ELISA is also used to test for the presence of IgG antibodies against the spike proteins of SARS-CoV-2 and MERS-CoV [26]. Luis G. Giménez-Lirola et al. developed an indirect ELISA capable of detecting ASFV antibodies in serum or oral fluid specimens [12]. Although ELISA is a traditional and stable test method, it has some shortcomings related to its complexity and time consumption. Therefore, fast detection methods need to be developed in future research.

In summary, a dual ELISA based on CD2v and p30 was preliminary established for distinguishing wild-type ASFV and ASFV-ΔCD2v infection. As far as we know, this is the first dual ELISA simultaneously targeting the ASFV glycosylated CD2v and phosphorylated p30 proteins for the detection of ASFV antibody. If this dual ELISA can be used to detect ASFV antibody in the future, it will represent an important diagnostic method to distinguish between wild-type ASFV- and ASFV-ΔCD2v-infected pigs in farms, which will play an important role in ASFV epidemiology study.

## Data Availability

All data generated and analyzed in this research are included in the article.
